# Effect of age on virtual reality-assisted subjective visual vertical and subjective visual horizontal at different head-tilt angles

**DOI:** 10.1016/j.bjorl.2022.10.001

**Published:** 2022-10-14

**Authors:** Ying Cheng, Yuzhong Zhang, Weijun Ma, Yanfei Chen, Qing Zhang, Min Xu

**Affiliations:** a Department of Otolaryngology Head and Neck Surgery, The Second Affiliated Hospital of Xi’an Jiaotong University, Xi’an, China; b Xinhua Hospital of Shanghai Jiao Tong University School of Medicine, Shanghai, China

**Keywords:** Subjective visual vertical, Subjective visual horizontal, Age, Virtual reality, Utricle

## Abstract

**Objective:**

Subjective Visual Vertical (SVV) and Subjective Visual Horizontal (SVH) values may reflect bilateral utricle asymmetry. Bilateral utricle static tension balance can be used to evaluate bilateral otolith lesions and otolith-related central neuropathy. Few studies have examined Virtual Reality (VR)-assisted SVV and SVH values at various head-tilt angles across age groups. The present study aimed to determine the effects of age on VR-assisted SVV and SVH values at different head-tilt angles.

**Methods:**

We divided 180 healthy subjects into 6 age groups (n = 30 in each group). VR-assisted SVV and SVH measurements were performed at 9 head-tilt angles (head held vertically, 0°; head tilted 30°, 45°, 60°, and 90° to the left/right) in the roll plane.

**Results:**

SVV and SVH values significantly differed with head-tilt angle (*p* < 0.05). No significant difference was detected in the SVH and SVV values between different age groups (*p* = 0.632 and *p* = 0.810, respectively), and no interaction between the age group and the head-tilt angle was found for the SVH and SVV values (*p* = 0.670 and *p* = 0.084, respectively).

**Conclusion:**

These results suggest that age may have little effect on VR-assisted SVV and SVH at different head-tilt angles. Therefore, VR-assisted SVV and SVH can be evaluated as an effective, fast, and simple way to evaluate utricle function.

**Level of Evidence:**

Level 4.

## Highlights

• VR-assisted SVV and SVH data at different head-tilt angles were established.
• Age may not affect VR-assisted SVV and SVH values at different head-tilt angles.
• VR-assisted SVV and SVH tests are an effective way to evaluate utricle function.

## Introduction

The human vestibular system functions to maintain the body’s center of mass over its base of support, and this allows us to see clearly while moving, make automatic postural adjustments to maintain posture and stability, and identify spatial orientations. The vestibular system integrates information from the visual system, the vestibular system, and the proprioceptive portion of the central nervous system.^1^ The vestibular system includes the utricle, saccule, and three semicircular canals, and provides sensory information about motion, head position, and spatial orientation. The utricle and saccule are otolith organs that are specialized to detect movement in the horizontal and vertical planes, respectively. Information about movement in these planes is used to maintain the body posture through the estimation of gravity and the perception of verticality.

The Subjective Visual Vertical (SVV) and Subjective Visual Horizontal (SVH) values are two parameters that reflect the estimation of gravity and the perception of verticality and horizontality in a dark environment without visual references. SVV and SVH estimations depend on the integration of a variety of sensory information. In 1970, Freidmann discovered that patients with a unilateral loss of vestibular function showed a significant deviation in their perception of gravity.^2^ The angle of an SVV or SVH deviation may reflect bilateral utricle asymmetry. Bilateral utricle static tension balance can be used to evaluate bilateral otolith lesions and otolith-related central neuropathy.^3^, ^4^, ^5^, ^6^, ^7^ SVV and SVH assessments have therefore been used with increasing frequency in the diagnosis of otolith disorders.^3^, ^8^ Unlike other methods of evaluating vestibular otolith function, SVV and SVH assessments can be performed without complex equipment. Moreover, the equipment used to measure SVV and SVH values is sensitive and easy to operate.

SVV and SVH assessments are usually performed under static conditions, with a line shown against a stationary black background.^9^ Virtual Reality (VR) systems can provide a black background that can be used for SVV and SVH assessments. However, few studies have examined VR-assisted SVV and SVH assessments at various head-tilt angles across different age groups. In this study, we performed VR-assisted SVV and SVH assessments at head-tilt angles of varying severity in healthy subjects of different ages. Our aim was to establish reference SVV and SVH data for different age groups and clarify the effect of age on SVV and SVH values.

## Methods

### Participants

Healthy family members of patients who were hospitalized at The Second Affiliated Hospital of Xi’an Jiaotong University between January 2019 and December 2021 and the medical staff at the same institution were enrolled in this study. The inclusion criteria were as follows: (1) No history of ear disease (e.g., otitis media and deafness), imbalance, vertigo, or injury to the nervous system, skeletal system, or head; (2) Good neck and upper limb function; (3) Normal vision or corrected visual acuity of logMAR ≥ 1.0 with lenses and no astigmatism; (4) Normal results on otoscopy, pure-tone audiometry, and acoustic impedance (almost-normal results were acceptable for elderly patients); and (5) Signed informed consent and cooperation with the examination. The exclusion criteria were as follows: (1) History of dizziness and vertigo, hearing abnormalities, or eye disease, and (2) Abnormal results on otoscopy, pure-tone audiometry, or acoustic impedance tests. Because of the difficulty in recruiting older subjects as well as natural changes in hearing threshold with age, the bounds of normal results for pure-tone audiometry were extended to ≤30 dB hearing loss for patients in the age groups of 50–60 years and ≥61 years.

### SVV and SVH measurements

**Figure 1:**
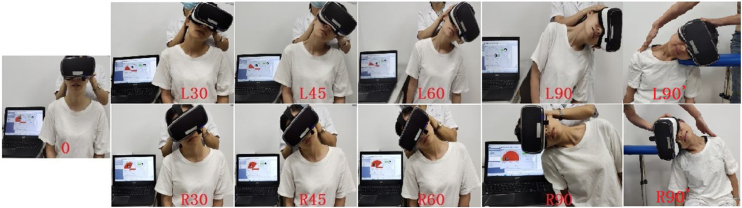
Schematic diagram showing the virtual reality-assisted measurement of SVV and SVH. (A) The state of the subject when using the virtual reality system. (B) Appearance of the visual field with the use of virtual reality glasses. Both the upper and lower views are optional. (C) The SVV and SVH detection system consists of virtual reality interactive goggles, a wireless control device, and PC software. (D) Reported form of SVV and SVH values. SVV, Subjective Visual Vertical; SVH, Subjective Visual Horizontal; PC, Personal Computer.

All measurements were performed by a specialist with 5 years of clinical experience. Each subject completed the SVV and SVH tasks at 9 head-tilt angles in the roll plane: (1) Head held vertically (0°), (2) Head tilted 30° to the left, (3) Head tilted 45° to the left, (4) Head tilted 60° to the left, (5) Head tilted 90° to the left, (6) Head tilted 30° to the right, (7) Head tilted 45° to the right, (8) Head tilted 60° to the right, and (9) Head tilted 90° to the right. Some subjects had difficulty in tilting their head to a 90° angle. These subjects were asked to rest their heads on the table and place their left/right cheek on the table to achieve a head tilt of 90° to the left/right (Fig. 1). The measurement process and study methodology were explained in detail to each subject before the measurements were performed. Two preliminary measurements were performed to familiarize the subjects with the measurement process. For each subject, measurements were performed 3 times at each head position; all 3 measurements for each head-tilt angle were performed one after the other, and the average values were calculated.

**Figure 2:**
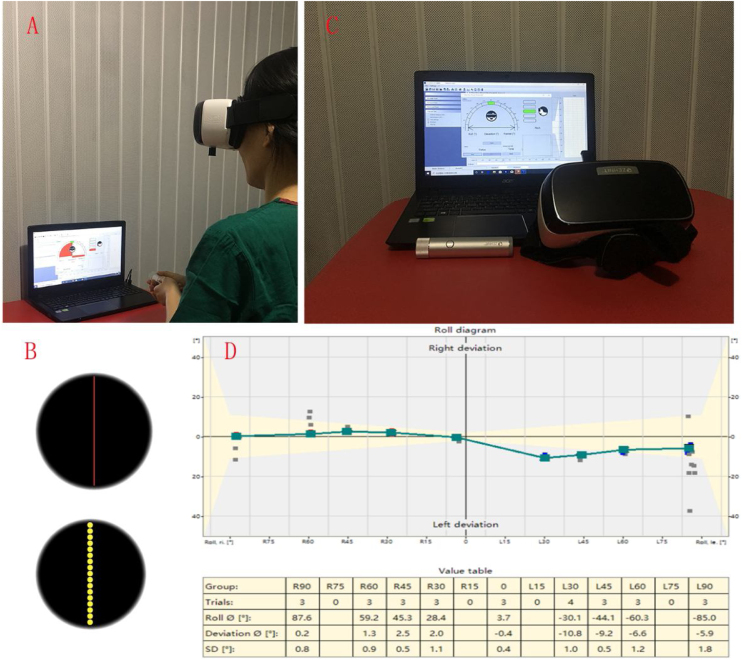
SVV and SVH at 9 head-tilt angles in the roll plane (0, L30, L45, L60, L90, R30, R45, R60, and R90). Some subjects had difficulty with performing a 90° head tilt. These subjects were advised to rest their heads on the table, with their left/right cheek on the table, to achieve a head tilt of 90° to the left/right (L90, R90). SVV, Subjective Visual Vertical; SVH, Subjective Visual Horizontal.

The SVV and SVH assessments were conducted using a VertiSVV SVV/SVH detection system (Balancecare, Shanghai, China). This system consists of VR goggles, a wireless control device, and Personal Computer (PC) software (Fig. 2C). It can detect SVV/SVH values with a detection accuracy of 0.1° and display the subject’s head position in real time (Fig. 2D). The subject sits in an upright position and enters a dark environment upon affixing the VR glasses (Fig. 2A). By means of the VR glasses, the subject will see a red bar of light or a light bar composed of several yellow-green light spots in the middle of a black background (Fig. 2B). We selected the yellow-green option for use in this study. The initial position of the light bar was randomly determined by the PC connected to the VR glasses. The subject adjusted the light bar to the desired position by using the wireless control device, and then pressed the confirmation button. The PC recorded the specific angle of the subject’s head and the angle of SVV/SVH deflection at a given time-point. The angle of the light bar was then adjusted for the next measurement. The magnitude of this adjustment was randomized. This process was repeated 3 times, and the values obtained were averaged. The vertical line of gravity was defined as an SVV of 0°. The horizontal line of gravity was defined as an SVH of 0°. The field of view was divided into 4 quadrants with the 2 gravity baselines as the vertical and horizontal coordinates. The SVV was considered to be positive (+) if its upper end was to the right of the vertical axis, and its lower end was to the left of the vertical axis. The SVV was considered to be negative (-) if its upper end was to the left of the vertical axis, and its lower end was to the right of the vertical axis. Similarly, the SVH was considered positive (+) if its left end was above the horizontal axis, and its right end was below the horizontal axis. The SVH was set as negative (-) if its left end was below the horizontal axis, and its right end was above the horizontal axis. The PC was used to measure the deviation of the SVV/SVH with respect to the vertical and horizontal lines of gravity (the deflection angle of the head in the roll plane). Head position was monitored in real time to ensure that the head remained in the required position throughout the study and to prevent deflection of the head in the pitch plane, which reduces the influence of the semicircular canal on the measurements (Fig. 2).

### Statistical analysis

The subjects were divided into 6 groups based on age: ≤20 years, 21–30 years, 31–40 years, 41–50 years, 51–60 years, and ≥ 61 years. The subjects were matched by gender across all age groups to reduce the influence of gender on the research findings. Statistical analysis was performed using SPSS version 18.0. Data with a normal distribution were analyzed using repeated-measures analysis of variance (ANOVA). The Greenhouse-Geisser method was used to estimate the coefficient of Mauchly’s test of sphericity and correct the *F*-value. All data are expressed as mean ± Standard Deviation (SD); *p* < 0.05 was considered statistically significant.

### Ethical considerations

This study was approved by the Ethical Review Committee of the Second Affiliated Hospital of Xi’an Jiaotong University. Written consent forms for the use of the subjects’ data were obtained from all subjects prior to their participation in the study.

## Results

### Demographic characteristics

A total of 180 subjects (90 males and 90 females) were enrolled in this study. The participants’ ages ranged from 7 to 71 years (mean: 39.48 ± 17.40 years). The subjects were divided into 6 age groups as follows: ≤20 years of age (n = 30, mean: 13.86 ± 4.61 years); 21–30 years of age (n = 30, mean: 25.73 ± 3.10 years); 31–40 years of age (n = 30, mean: 34.20 ± 2.54 years); 41–50 years of age (n = 30; mean: 44.20 ± 2.69 years); 51–60 years of age (n = 30, mean: 54.20 ± 2.48 years); and ≥61 years (n = 30; mean: 64.70 ± 3.17 years).

### SVV and SVH values at different head-tilt angles

The SVV and SVH values were normally distributed at all head-tilt angles in all age groups. All the SVV and SVH data are presented as mean and SD, and minimum and maximum values in Table 1, Table 2.

**Table 1:** SVV and SVH values at different head-tilt angles in different age groups.

Head-tilt angle (°)	≤20 yrs (n = 30)	21–30 yrs (n = 30)	31–40 yrs (n = 30)	41–50 yrs (n = 30)	51–60 yrs (n = 30)	≥61 yrs (n = 30)
	**SVV (°)**					
90R	1.97 ± 10.82	4.30 ± 6.23	2.75 ± 5.04	2.42 ± 6.74	0.36 ± 9.44	3.91 ± 10.40
60R	3.32 ± 9.50	0.18 ± 8.85	−2.52 ± 6.64	−1.25 ± 7.48	−0.87 ± 11.39	−0.58 ± 8.12
45R	−0.86 ± 10.06	−1.69 ± 7.98	−0.01 ± 5.77	−0.58 ± 7.32	−1.75 ± 7.94	2.65 ± 11.71
30R	−0.34 ± 9.14	0.43 ± 6.50	0.10 ± 5.17	−0.46 ± 5.84	−1.80 ± 8.97	3.85 ± 7.20
0	2.19 ± 10.96	−0.59 ± 6.13	−0.69 ± 5.16	−0.01 ± 4.95	−1.52 ± 8.35	−4.58 ± 10.58
30L	0.97 ± 11.49	−0.94 ± 6.15	−0.43 ± 6.68	−2.88 ± 5.37	−2.43 ± 8.61	−0.78 ± 9.78
45L	−0.49 ± 12.19	−0.75 ± 7.31	0.25 ± 7.19	−0.69 ± 8.60	−1.64 ± 9.78	−2.53 ± 9.72
60L	−0.16 ± 10.00	−0.67 ± 6.53	1.30 ± 5.71	−0.90 ± 7.62	3.12 ± 9.83	−1.40 ± 10.58
90L	−0.07 ± 10.46	−3.24 ± 6.25	−0.35 ± 8.40	−2.80 ± 7.85	−2.20 ± 10.69	−4.00 ± 11.89
	**SVH (°)**					
90R	2.88 ± 9.45	0.28 ± 8.00	2.29 ± 6.20	2.94 ± 7.30	0.02 ± 9.51	1.09 ± 10.72
60R	−2.23 ± 10.08	−1.11 ± 7.54	−3.15 ± 6.03	−3.50 ± 6.92	−4.94 ± 8.91	−2.61 ± 11.44
45R	−0.13 ± 10.82	−0.75 ± 7.62	−2.67 ± 7.27	−0.94 ± 7.41	−2.40 ± 8.76	−0.33 ± 9.97
30R	−2.73 ± 11.29	−2.04 ± 7.33	−1.28 ± 5.97	0.78 ± 7.16	0.72 ± 9.25	1.44 ± 8.54
0	1.07 ± 10.72	−1.42 ± 6.61	−1.00 ± 6.38	0.37 ± 5.50	2.05 ± 8.13	−2.20 ± 6.23
30L	−0.51 ± 11.18	−2.12 ± 6.54	0.81 ± 4.77	−0.40 ± 7.18	1.70 ± 8.40	−1.43 ± 9.57
45L	2.04 ± 11.56	0.02 ± 6.49	1.04 ± 5.81	2.43 ± 7.11	0.52 ± 8.61	0.07 ± 9.10
60L	2.26 ± 8.88	−1.86 ± 7.44	−1.58 ± 5.70	0.88 ± 7.80	2.26 ± 9.40	−1.14 ± 9.58
90L	−2.45 ± 11.42	−1.88 ± 7.91	−1.23 ± 7.56	−1.99 ± 6.67	−4.14 ± 11.12	−0.22 ± 13.13

SVV, Subjective Visual Vertical; SVH, Subjective Visual Horizontal; R, Right; L, Left.

The values shown are expressed as mean and standard deviation.

**Table 2:** Minimum and maximum SVV and SVH values at different head-tilt angles in different age groups.

Head-tilt angle (°)	≤ 20 yrs (n = 30)		21–30 yrs (n = 30)		31–40 yrs (n = 30)		41–50 yrs (n = 30)		51–60 yrs (n = 30)		≥ 61 yrs (n = 30)	
	Min	Max	Min	Max	Min	Max	Min	Max	Min	Max	Min	Max
	**SVV (°)**											
90R	7.00	19.4	−11.8	12.5	−11.6	11.6	−12.8	13.6	−17.4	19.1	−18.4	21.1
60R	−18.6	14.9	−19.5	12.4	−11.5	11.5	−12.1	14.1	−17.0	19.1	−21.0	20.7
45R	−18.0	15.8	−18.9	10.9	−12.4	10.2	−13.9	13.3	−18.4	13.5	−20.6	20.3
30R	−18.7	18.6	−11.0	12.0	−10.5	10.3	−13.6	10.1	−16.9	16.9	−5.2	18.6
0	−17.7	19.2	−12.5	12.0	−10.5	11.4	−10.3	10.2	−18.5	18.1	−20.4	18.7
30L	−17.9	18.4	−11.9	10.6	−11.0	11.0	−11.6	10.3	−17.8	18.7	−18.1	17.6
45L	−20.0	18.2	−13.6	12.4	−11.5	11.5	−12.8	16.6	−18.1	16.2	−19.8	20.1
60L	−19.4	16.5	−11.0	11.0	−11.1	10.4	−10.3	12.5	−13.8	18.6	−18.0	21.1
90L	−15.2	17.6	−14.3	11.0	−21.1	11.5	−14.9	12.3	−17.9	17.1	−28.2	16.6
	**SVH (°)**											
90R	−19.2	17.8	−12.4	15.0	−10.8	11.4	−13.2	15.5	−18.9	15.3	−20.0	22.7
60R	−18.0	17.4	−14.6	10.9	−13.5	10.8	−13.5	13.4	−18.9	11.3	−23.2	21.8
45R	−20.6	18.4	−12.1	12.3	−14.9	11.1	−13.0	14.4	−16.8	13.1	−22.3	18.3
30R	−17.9	17.6	−12.3	12.4	−11.8	11.4	−16.5	18.4	−17.7	16.9	−16.0	17.4
0	−22.8	19.4	−12.3	12.3	−11.3	10.6	−8.9	13.3	−17.3	18.5	−12.5	17.7
30L	−18.2	18.5	−11.7	12.2	−7.4	10.1	−14.6	12.1	−15.2	19.1	−19.5	15.3
45L	−18.7	17.5	−10.1	12.4	−10.6	11.2	−11.3	15.0	−15.6	15.0	−19.0	16.5
60L	−17.9	19.4	−17.0	10.7	−9.8	8.4	−11.5	17.9	−16.6	17.8	−21.9	15.2
90L	−28.8	17.7	−15.1	12.4	−13.7	11.3	−12.9	9.9	−19.6	18.2	−20.5	20.9

SVV, Subjective Visual Vertical; SVH, Subjective Visual Horizontal; R, Right; L, Left.

### Comparison of SVV and SVH values among age groups

For each subject, the SVV and SVH values were separately measured at different head-tilt angles. We found that the effect of age on the SVV and SVH values at different angles was not completely independent. The SVV and SVH values did not comply with the “symmetry” hypothesis, and thus, they were compared using repeated-measures ANOVA. The Greenhouse-Geisser method was used to estimate the coefficient of Mauchly’s test of sphericity and correct the F-value. We found statistically significant differences in the SVV and SVH values at different head-tilt angles (*p* < 0.05; Table 3). No significant difference was detected in the SVH and SVV values between different age groups (*p* = 0.632 and *p* = 0.810, respectively), and no interaction between the age group and the head-tilt angle was found for the SVH and SVV values (*p* = 0.670 and *p* = 0.084, respectively).

**Table 3:** Variance in SVV and SVH values at different head-tilt angles across different age groups.

Factors	Sum of squares	*df*	Mean square	*F*	p
	**SVV**				
Head-tilt angle	2002.328	6.905	290.001	3.323	**0.002**
Age	249.056	5	49.811	0.689	0.632
Head-tilt angle * age interaction	2647.631	34.523	76.692	0.879	0.670
	**SVH**				
Head-tilt angle	1452.140	7.141	203.351	2.505	**0.014**
Age	185.800	5	37.160	0.454	0.810
Head-tilt angle * age interaction	3909.128	35.705	109.483	1.349	0.084

SVV, Subjective Visual Vertical; SVH, Subjective Visual Horizontal.

*p*-values shown in bold are significant.

## Discussion

In the present study, we measured the SVV and SVH values in healthy subjects belonging to various age groups by using a VR-assisted system. Our data showed no age-related differences in the SVV and SVH values at different head-tilt angles.

The vestibular system is an important part of the human body’s sense of balance. In the utricle, planar polarity and hair cell organization contribute to the sensation of linear acceleration in the ground plane, in all directions and for all changes of head position.^10^ At present, the measurement of SVV and SVH values during static head tilt (roll plane) depends on the asymmetry of the information input from the right and left otoliths. This results in misjudgment of the gravity line by the cerebral cortex and in deflection of the gravity line because of the changes in eye position induced by the ocular reflex path. This latter mechanism is of primary importance. When the individual is in a static state, tilting the head to one side of the shoulder can cause reverse compensatory torsional eye movements, which are referred to as ocular counter-rolling. This response is known as the vestibulo-ocular reflex and depends on the utricle. Because special equipment is needed to observe rolling eye movements, SVV and SVH measurements are widely used in clinical practice as simple, reliable methods with which to evaluate the vestibular utricle system.^11^, ^12^ SVV and SVH assessments are also used to evaluate peripheral and central spatial disorientation in patients with vestibular disorders.^13^

Studies have found degradation of vestibular hair cell function and decreased numbers of vestibular ganglion cells, vestibular nerve fibers, and vestibular nerve cells among elderly patients.^14^, ^15^ Furthermore, vestibular function testing has provided evidence of reduced vestibular system function in elderly patients, as compared with younger patients.^16^ However, some studies have reported conflicting results. In some cases, SVV tests revealed no significant differences between elderly and younger patients.^17^, ^18^ In a study of patients with temporal bone pathology, Igarashi et al. found no differences in utricle otolith volume between children aged 2–2.5 years and adults aged 58–87 years.^19^ Rosenhall et al. compared elderly patients with temporal bone pathology to younger individuals; the results showed that among older patients, the number of ampulla hair cells in the semicircular canals had decreased by 40% and the number of hair cells in the utricle had decreased by 21% relative to the younger patients.^14^ Based on these results, one might expect elderly patients to have decreased scores on the head impulse test, whereas SVV and SVH values may be less affected by age.^20^ Therefore, SVV and SVH assessments may be a good way to evaluate vestibular function in the elderly. Our data showed that the SVV and SVH values at various head-tilt angles were similar across all the age groups examined. These findings are consistent with those reported previously.^15^, ^18^ Kullmann et al. analyzed data from 290 older pediatric patients (late adolescents; 18–21 years of age) and 449 adult patients (21–45 years of age), and found that age had no effect on SVV or SVH values when the head remained in the upright position.^21^ Ferreira et al. evaluated 100 healthy volunteers of ages 18 to 59 years, and found that the SVV values, as evaluated using the bucket method, did not increase with age in healthy Brazilian individuals.^22^ Toupet et al. found that the cumulative SVV tilt did not progress with iteration among patients aged between 20 and 49 years, but did do so in younger and extremely older age groups (i.e., 0–19 years and >80 years).^23^ In our study, all the subjects were younger than 80 years.

This study has some limitations. We were unable to recruit persons aged >71 years, so our results are not representative of older age groups. We therefore suggest that the range of normal values for various age groups be established at each research unit, in order to evaluate abnormal results most accurately.

## Conclusion

We have established a normative database of VR-assisted SVV and SVH values at different head-tilt angles for all age groups. Neither VR-assisted SVV nor SVH was affected by age at any of the head-tilt angles examined. These results suggest that VR-assisted SVV and SVH can be evaluated as an effective, fast, and simple way to assess utricle function in persons aged below 70 years.

## Authors’ contributions

M.X., Q.Z. and Y.C. contributed to conceptualization, funding acquisition and project administration of the study. Methodology, resources were, and software were performed by Y.C., Y.Z., W.M. and Y.F.C. Supervision and validation were performed by Q.Z. and M.X. Visualization and Writing-original draft was performed by Y.C. and all authors commented on writing-review and editing.

## Abbreviations

VR, Virtual Reality; SVV, Subjective Visual Vertical; SVH, Subjective Visual Horizontal; PC, Personal Computer; SD, Standard Deviation.

## Data availability

The datasets generated during and/or analysed during the current study are available from the corresponding author on reasonable request.

## Statement of ethics

This study was approved by the Ethical Review Committee of the Second Affiliated Hospital of Xi'an Jiaotong University (2021-858). Written consent forms for the use of subject data were obtained from all subjects prior to their participation in the study.

## Funding

This work was supported by Key r＆d projects in Shaanxi Province, China (nº 2018SF-189); National Natural Science Foundation of China (NSFC) (81970891). The funders had no role in the research design and result interpretation.

## Conflicts of interest

The authors declare no conflicts of interest.
